# Significance of Hormone Alteration Following Bariatric Surgery

**DOI:** 10.7759/cureus.49053

**Published:** 2023-11-19

**Authors:** Ethan Slouha, Enas M Elkersh, Allison Shay, Shanalyn Ghosh, Aisha Mahmood, Vasavi R Gorantla

**Affiliations:** 1 Anatomical Sciences, School of Medicine, St. George's University, True Blue, GRD; 2 Anatomical Sciences, School of Medicine, St. George's University, St. George's, GRD; 3 Anatomical Sciences, School of Medicine, St. George's University, St.George's, GRD; 4 Biomedical Sciences, West Virginia School of Osteopathic Medicine, Lewisburg, USA

**Keywords:** pyy, adiponectin, leptin, glp-1, ghrelin, laparoscopic adjustable gastric band, gastric sleeve, roux-en y bypass, bariatric surgery

## Abstract

The prevalence of obesity has increased significantly over the last several decades, and with its increase comes a wide variety of comorbidities, such as diabetes and cardiovascular disease. Traditionally, diet and exercise have been prescribed for individuals to try and regain control of their weight and health status. Despite this successful method, the compliance rate is significantly below the desired amount. Over the last few decades, a new treatment has been offered to significantly decrease an individual's weight to an optimal BMI between 18 and 25 kg/m^2^. Bariatric surgery has been proposed to be the most appropriate treatment for obesity, and there are several different types of bariatric surgery: Roux-en-Y gastric bypass (RYGB), biliopancreatic diversion with duodenal switch (BPD-DS), adjustable gastric band (AGB), and sleeve gastrectomy (SG). Hormones may be significantly involved in losing and maintaining weight loss. This paper aims to evaluate hormone changes in appetite suppression, appetite activation, glycemic control, and lipid metabolism and how these impact overall weight loss concerning the most prominent surgeries. The hormones assessed were ghrelin, insulin, leptin, GLP-1, PYY, and adiponectin, and their levels before and after each surgery were compared. RYGB is one of the most successful types of bariatric surgeries, and this correlates with it having the most suppressed levels of ghrelin, insulin, and leptin following surgery with a slow return to normal. RYGB has also led to the most significant increased levels of PYY, pre- and post-prandial GLP-1, and adiponectin. Hormones following SG followed the hormone trend after RYGB but were not as prominent. BDP-DS has the highest success rate. However, numerous adverse effects have limited the amount of studies assessing the surgery. What was present was not as significant as RYGB, possibly due to manipulation.

## Introduction and background

Obesity has reached epidemic proportions worldwide, accounting for more than 1.9 billion overweight and approximately 650 million obese adults [1]. There is greater morbidity and mortality in patients with severe obesity, especially in patients with a BMI > 35 kg/m^2^ [1]. Obesity is often associated with comorbidities such as type 2 diabetes mellitus (T2DM), coronary heart disease, hypertension, dyslipidemia, stroke, and some cancers [1]. Bariatric surgery has become popular in treating obesity, where patients cannot lose weight via traditional methods such as diet and exercise or who have a BMI ≥ 35 kg/m^2^ with comorbidities [2]. Most bariatric surgeries alter the gastrointestinal tract anatomy, which alters hormone levels.

Restrictive and malabsorptive surgeries

The biliopancreatic diversion with duodenal switch surgery (BPD-DS) combines restrictive and malabsorptive mechanisms by creating a 250 cm^3^ sleeve gastrectomy while the duodenum is transected 4 cm distal to the pylorus [3-5]. This procedure has a long-term success rate of around 92% [6]. For morbidly obese patients, one of the most common procedures for weight loss is a gastric bypass (RYGB) or laparoscopic RYGB (LRYGB), with about an 85% success rate of maintaining 50% weight loss for more than five years [7]. In RYGB, a small abdominal pouch is connected to the small intestine. Ingested food goes directly into the stomach's small pouch and the small intestine, bypassing most of the stomach and duodenum [8]. This method is a restrictive and malabsorptive procedure, thus causing significant weight loss by decreasing energy intake and consumption of nutrients [9,10].

Restrictive surgeries

SG is a vertical gastrectomy that creates a complete separation and removal of the greater curvature of the stomach, preserving the pylorus, a longitudinal resection of the fundus, and antrum, together forming a tubular duct along the lesser curvature [11,12]. The stomach size is reduced to 75-80% of the original without significant anatomical changes and has a long-term success rate of 80% [11,13-15]. Adjustable gastric band (AGB) was created in 1993 and modified to current standards with a 70% success rate [14,16]. The AGB is a laparoscopic procedure that requires the insertion of trocars through incisions to allow for the opening of the peritoneum from the fundus to the diaphragm of the stomach [17]. A calibration tube inserted into the stomach determines the band's positioning before being attached to a subcutaneous port where the saline will flow upon inflation [17].

Important hormones that influence satiety after bariatric surgery

Ghrelin is an orexigenic hormone secreted chiefly from the gastric fundus by oxyntic gland endocrine cells that increases energy intake by stimulating hunger [18,19]. Increasing ghrelin concentrations increases lipogenesis and adipogenesis while decreasing lipolysis, leading to obesity and insulin resistance; this can be seen after weight loss [19-21]. Insulin and weight have a unique cycle; obese individuals develop insulin resistance and T2DM over time due to the excess intake of food rich in carbohydrates and fat [22]. Before treatment, this insulin sensitivity leads to a loss in weight through insulin's effects on lipolysis [22]. Once treatment is started on T2DM patients, weight increases [22]. This is termed "catch-up" regain as initially, patients with T2DM lose weight during the initial decrease in insulin after the disease sets in [22,23]. Ideally, a reduction in insulin following bariatric surgery would also contribute to weight loss.

Leptin is primarily produced by adipose tissue and, in lesser amounts, the mammary epithelium, heart, and stomach [24]. Leptin concentrations are correlated positively with the amount of fat in the body [25]. Obese individuals develop leptin resistance, leading to overconsumption of food while maintaining high levels of leptin [26]. GLP-1 and PYY are released from L cells primarily in the ileum and influence satiety and glycemic homeostasis [27,28]. GLP-1 is an incretin hormone influencing insulin release, and PYY (PYY3-36) contributes to gastric emptying and directly affects satiety [29,30]. Adiponectin has beneficial effects on lipid and glucose metabolism, inflammation, insulin sensitivity, and vascular homeostasis; an increase in adiponectin has been associated with reducing LDL-cholesterol and improving HDL [31].

This review highlights the hormonal changes following each bariatric surgery and compares them to the failure rates. There are many stipulations about why these bariatric surgeries fail, including psychiatric complications, lack of compliance, and intolerance. No articles covered the hormonal aspects of weight loss following bariatric surgery. Hormones make moderate to drastic changes to the long-term gut-hormone axis. This paper describes the relationship between hormonal change and success rate in different bariatric surgeries that can be used for future experimental research to reduce weight regain.

## Review

Methods

A comprehensive literature review was done using ScienceDirect, PubMed, and ProQuest databases from January 1, 2002, to December 31, 2022. Keywords included ‘hormones and biliopancreatic diversion with duodenal switch,’ ‘hormones and Roux-en-Y gastric bypass,’ ‘hormones and adjustable gastric band,’ ‘hormones and sleeve gastrectomy,’ and ‘hormones and one-anastomosis/mini-gastric bypass.’ Our electronic search focused on peer-reviewed articles in line with the objective of this paper. Articles published before 1992, duplicates, and articles not written in English were excluded during the first step of the screening process. Once the search was complete, five coauthors reviewed the papers independently. Articles selected were analyzed based on the publication's title, abstract, study type, and full-text accessibility. Our initial search resulted in 10,846 articles. These articles were further filtered using keywords, abstraction, inclusion, and exclusion criteria. Ultimately, 49 articles were found to be within our scope.

Inclusion Criteria

The following inclusion criteria were used: articles published between 1992 and 2022, articles relevant to hormone alterations following bariatric surgery, articles written in English, articles with full-text accessibility, peer review, and included case-control, observational, cohort, and meta-analysis on humans.

Exclusion criteria

**Figure 1:**
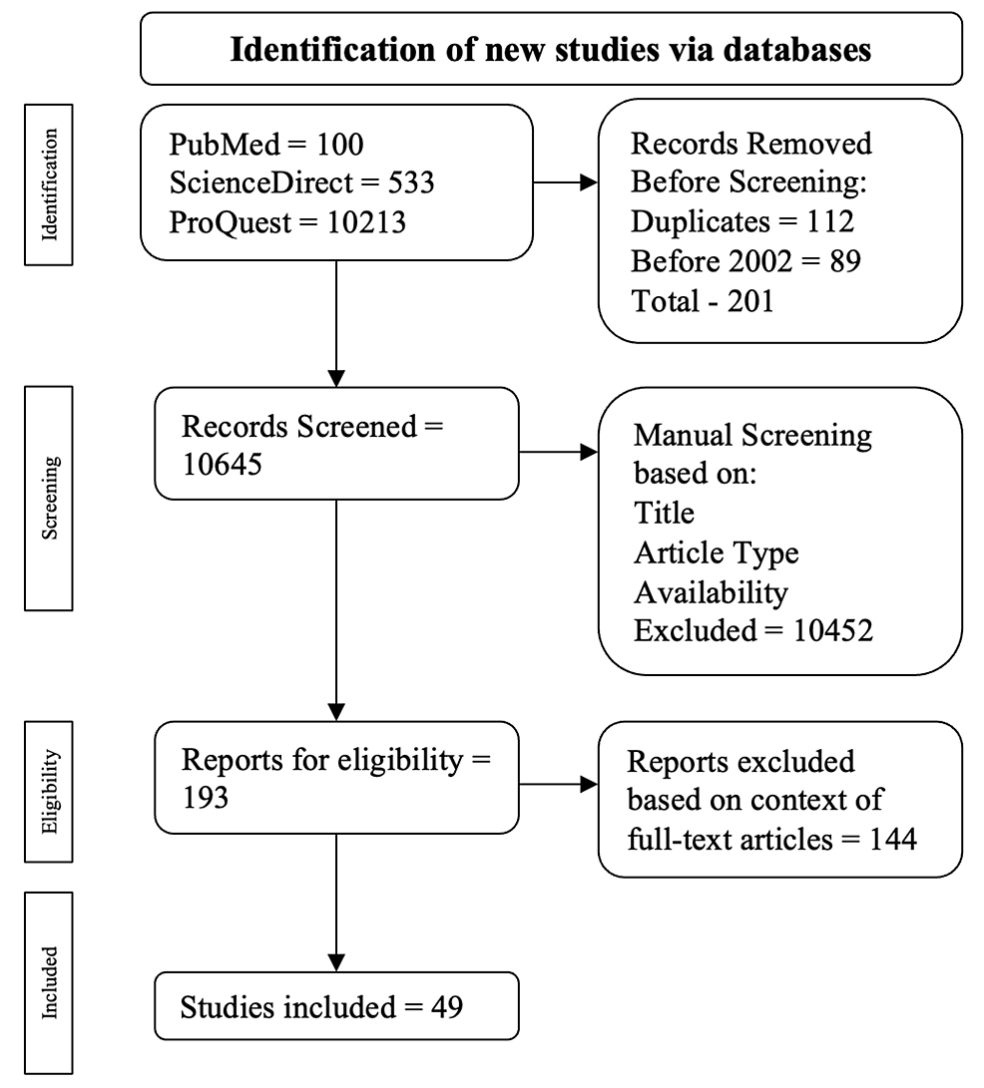
Flow of the article analysis based on exclusion and inclusion criteria for this systematic review The process for this review was done as described in the PRISMA statement [32]

The following exclusion criteria were used: animal studies, systematic reviews, review articles, and case reports. Duplicates were also excluded. The inclusion and exclusion process of the articles is illustrated in Figure 1.

Bias

All studies were assessed for bias. A medium risk for bias was determined as most studies were done prospectively based on lab results and controlled factors. Grading of Recommendation, Assessments, Development, and Evaluation (GRADE) was used to assess individual studies for bias. GRADE is a tool to evaluate flaws such as indirectness, imprecision, and publications.

Results

In total, 10,846 articles were found: 10,213 from ProQuest, 533 from ScienceDirect, and 100 from PudMed. Among the exclusions were 112 duplicates, and 89 papers published before 1992. This resulted in 201 articles being excluded from our search, leaving 193 articles for manual screening. Articles were manually screened based on their title, abstract, study type, and availability; 193 were checked for eligibility. Ultimately, 49 articles were picked, excluding articles without the full text.

BPD-DS is the most successful bariatric surgery. However, few studies were found concerning hormone alteration, probably due to the numerous short- and long-term adverse effects. The results of the studies were still compared to the other surgeries. In comparison, it was observed that both RYGB and SG produced the most significant suppression in ghrelin, insulin, and leptin levels compared to AGB and BPD-DS. RYGB and SG also significantly increased in PYY, pre- and postprandial GLP-1, and adiponectin. However, RYGB proved superior in hormonal alterations than SG, which may correlate with other studies indicating RYGB to be the most successful bariatric surgery.

Discussion

Ghrelin

Despite having a 90% success rate, relatively few studies evaluated the hormone alterations following BPD-DS, and only three studies specifically analyzed the ghrelin concentrations. Following BPD-DS, ghrelin concentrations significantly decreased, correlating with increased satiety in all three studies [33-35]. Following RYGB, most studies found that total plasma ghrelin concentrations were significantly suppressed [36-38]. One study found that ghrelin concentrations increased in the months following RYBG but stayed significantly less than the control group throughout the experiment [39]. Two studies observed that ghrelin concentrations remained unchanged/slightly decreased initially but increased afterward [40,41]. Fast and acyl-ghrelin concentrations, in several studies, were not significantly changed initially and up to 12 months [42-48]. This was contradicted in three studies that observed significantly increased acylated and plasma ghrelin concentrations compared to baseline following RYGB [41,49,50].

Dirksen et al. compared the ghrelin concentrations of those who responded well to weight loss following RYGB and those who did not and found that fasting ghrelin concentrations were lowest in poor responders. In contrast, postprandial ghrelin suppression was significantly higher in good responders [51]. Christou et al. contradicted these results, who found no correlation between plasma ghrelin concentrations of patients with successful weight loss and those who failed to accomplish weight loss [52]. In the long term, ghrelin concentrations did not decrease with the decrease in weight, revealing that ghrelin concentrations may not be affected by weight loss following RYGB [50,53].

Pre- and postprandial ghrelin concentrations decreased significantly in SG patients at all time points [40,44,54]. Contrary to most of these studies, Kalinowski et al. found that fasting ghrelin concentrations were significantly decreased at one month following SG but recovered non-significantly at six months and remained comparable to baseline until 12 months [43]. It was observed that there was no correlation was found between BMI and ghrelin at 12 months following SG [50]. However, Lampropoulos et al. found a significant decrease in postprandial ghrelin in the weight maintenance group compared to the weight regain group [55]. Despite a significant decrease in ghrelin observed in the studies, a reduction of postprandial hunger at 52 weeks was not maintained after SG [56]. In AGB, both fundic, acylated, and unacylated plasma ghrelin were lower in morbidly obese patients but significantly increased six months postoperatively [36,39,41,45]. Fasting ghrelin concentrations did not increase significantly 52 weeks after AGB, but there was a significant change in plasma ghrelin postprandially [45]. Dixon et al. found a non-significant increasing trend in ghrelin concentrations compared to controls without surgery [57]. Most studies observed that ghrelin concentrations returned or stayed close to baseline/control values [36,57,58].

When comparing the surgeries, it was observed that one-week following RYGB and SG, ghrelin concentrations were significantly lower than pre-operative values and were more significantly decreased after SG [37,42,43,47,49,56,59,60]. However, Benedix et al. found that ghrelin concentrations varied between 6 to 12 months after SG and RYGB, initially decreasing and, by 12 months, increased higher than the baseline readings in SG compared to RYGB [58]. Lee et al. found that preprandial des-acyl ghrelin and postprandial acyl ghrelin were significantly decreased in RYGB patients compared to SG patients [46]. Considering these studies and their varying successes, ghrelin plays a role in weight maintenance but is not the only factor.

Insulin

The limited studies involving insulin analysis following BPD-DS all found that insulin was significantly reduced compared to controls [35,61,62]. Michaud et al. found that fasting insulin concentrations significantly reduced three days following BPD-DS [35]. Following RYGB, all studies corroborated that pre-/postprandial and overall insulin concentrations decreased significantly following surgery [38,43,45,54,56,58,63-67]. Plasma proinsulin concentrations were similarly lowered with modifications of insulin [65].

Insulin response following SG was like the other surgeries mentioned, with a significant decrease in fasting insulin concentrations [35,43,47,54,56,66]. Yang et al. observed that this was true at every sampled time interval in the 12-month duration of their study [47]. Following AGB, most studies observed that insulin concentrations decreased significantly compared to baseline and controls [57,63,68]. Two studies found the insulin decrease insignificant [45,58].

Comparing ABG, SG, and RYGB, there was a decrease in preprandial insulin concentrations, but only significant following SG and RYGB [58]. Gu et al. observed that the significant decrease in insulin following BPD-D, RYGB, and SG was not different from each other [35,49]. This was countered by Gomez-Martin et al., who observed that circulating preprandial insulin concentrations decreased more prominently after RYGB than SG [64]. Korner et al. observed that the decrease in insulin following surgery was significant at 52 weeks only after RYGB and not AGB [45].

Leptin

Only four studies were found for BPD-DS analyzing the leptin profile following surgery. There is some variation between these four studies. Kotidis et al. saw no significant change in leptin concentrations following BPD-DS [34]. While two other studies found that following BPD-DS, mean leptin concentrations decreased in the first year and remained stable throughout the study [33,69]. In line with that study, Elias et al. found that leptin concentrations were more than halved following surgery and were negatively correlated with weight loss [33]. Studies on leptin concentrations following RYGB stated that leptin decreased following RYGB [43,45,48,66,70,71]. Seridi et al. found that leptin concentrations immediately declined after RYGB, whereas the majority observed a decrease one week following RYGB and progressively dropped with weight loss [43,48,66,71]. Edwards et al. found a 2.7-fold decrease in leptin concentrations [70]. Weight loss was significantly correlated with leptin concentrations in the RYGB [45].

Like with RYGB, studies on SG were consistent in their results of leptin concentrations being significantly decreased [43,48,49,66,70,72]. Studies assessing leptin concentrations following AGB were not as decisive as the other surgeries discussed. Two studies observed a significant increase in leptin, up to a 1.6-fold increase, with no significant changes in body weight or fat [68,70]. On the other hand, one study found a decrease in leptin concentrations following AGB compared to the control, but this was not significant [57]. The last two studies found that leptin concentrations decreased significantly following AGB [45,60]. Korner et al. observed that weight loss was significantly correlated with leptin concentrations following AGB [45].

Of the studies included, RYGB produced the lowest leptin concentrations compared to SG, AGB, and BPD-DS. There was also a significant decrease in fasting and meal-induced leptin concentrations in SG and RYGB but significantly more in RYGB [41,49,54,56]. Compared to AGB alone, there was a significant decrease in leptin concentrations post-RYGB [45].

PYY-1

Again, there were limited studies on the hormone alterations following BPD-DS. Our extensive search found two studies observing that PYY concentrations significantly increased following BPD-DS [33,73]. Fasting PYY concentrations were higher after BPD-DS than controls and were continuously higher throughout the study [73]. Most RYGB studies observed a significant increase in postprandial PYY following RYGB [48,56,59,74]. Two studies analyzed PYY3-36 and found a significant increase in postprandial concentrations following RYGB [48,74]. In contrast, two other studies observed that three months following RYGB, post-prandial PYY concentrations were higher than baseline but were not statistically significant [54,74]. Some conflicting results were found concerning preprandial concentrations of PYY and PYY3-36. Four studies observed that preprandial PYY and PYYY-36 concentrations were significantly higher, whereas two other studies found no significant change [47-49,51,56,59]. One study did note that preprandial PPY decreased after RYGB with an exaggerated response 1 week after the operations [37]. These findings were non-significant at 3 months and 1 year following the surgery [37]

Like with RYGB, there was a consensus on increasing postprandial PYY concentrations following SG [40,48,75]. Peterli et al. found that preprandial PYY concentrations decreased but showed an exaggerated postprandial response one week after surgery. However, this was not replicable at three months or one year [37]. Three studies found no significant change in preprandial PYY and PYY3-36 concentrations following SG [48,49,56]. Lampropoulus et al. observed that PYY response levels showed an increase trend postprandially in weight regain patients who underwent SG [55]. Only two studies analyzed PYY concentrations following ABG, and they observed that postprandial and plasma PYY concentrations significantly increased in patients who underwent AGB [45,59].

Comparing surgeries, fasting PYY concentrations were increased significantly after both SG and RYGB, but at 12 months, they were higher in RYGB patients [47,49]. Lee et al. observed that postprandial PYY concentrations were increased following SG and RYGB at 30 and 60 minutes. Still, only concentrations in SG showed a unique decrease pattern at 90 minutes [46]. Compared to AGB, PYY concentrations significantly increased in the RYGB [45].

GLP-1

While limited research has been done on the effects of BPD-DS on GLP-1, there is a consensus that GLP-1 concentrations were significantly increased [33,35]. Cazzo et al. observed that after BPD-DS, GLP-1 concentrations were negatively correlated with the feeling of hunger, which may indicate involvement in successfully losing weight [76]. There was a little discrepancy in preprandial GLP-1 concentrations following RYGB, with most studies observing no significant change [40,47,48, 54,56,59,74]. Postprandial GLP-1, however, had a unified agreement that there was an increase following RYGB [37,38,40,46,48,54,59,65,74,77]. Interestingly, preprandial GLP-1 concentrations showed no changes between failed and sustained weight lost but postprandial GLP-1 concentrations were increased in sustained weight loss [51,78].

Unlike RYGB, there was no conflict between observations on preprandial GLP-1 concentrations following SG, as they showed no concentration changes [48,56]. The postprandial concentration of GLP-1 had conflicting observations, but most studies observed it significantly increased [37,40,48,56,75,79]. However, Arakawa et al. observed that it was not maintained at 52 weeks [56]. Only one study analyzed GLP-1 following AGB and found no concentration changes [77].

Compared to SG and AGB, RYGB seemed to have significantly higher pre- and postprandial GLP-1 [47,49,54]. One study, however, found that SG and RYGB patients have an increase in GLP-1 concentrations with no difference 2 years after surgery [46]. Compared to AGB, postprandial GLP-1 concentrations were three-fold greater in RYGB patients [45]. McCarty et al. speculate that the increase in GLP-1 in both RYGB and SG may be due to an improvement in the glycemic index [40].

Adiponectin

While limited studies were found, there were some discrepancies in the findings of how BPD-DS affects adiponectin concentrations. Both studies found non-significant but opposite trends of adiponectin following surgery [34,69]. Adami et al. found that changes in adiponectin concentrations were only significant in patients who were T2DM [69]. Studies following RYGB were consistent in their observations, showing increased adiponectin following surgery [58,64,66,80]. Benedix et al. found that over 12 months following RYGB, there was a significant increase in adiponectin concentrations up to 105% [58]. One study showed a correlation between increased circulating adiponectin and weight loss following RYGB [66].

Like RYGB, studies observed a significant increase in adiponectin concentrations following SG [64,66,72,80]. Shimizu et al. observed that at one month following SG, there was an increasing trend in adiponectin concentration that was significantly increased at six months [72]. One study observed that adiponectin was increased by 104% following SG [58]. Woelnerhanssen et al. observed that circulating adiponectin concentrations were significantly increased in correlation to weight loss following SG [66]. One study observed adiponectin following AGB, but both found that adiponectin significantly increased to 71.3% [58].

Comparison

Only two studies were found comparing adiponectin concentrations between surgery. One study observed that circulating adiponectin concentrations increased after surgery but were more prominent in RYGB than SG [64]. Michaud et al. observed no significant difference between BPD-DS and SG [35].

There are some limitations to this study, however. We did not analyze the psychological impact of bariatric surgery and how it influences eating. It is important to remember that surgery is an invasive treatment, and dietary restriction affects psychological eating disorders present before the surgery. This influences the long-term success rate of bariatric surgery. This systematic review does not include chemical effects on the brain and focuses solely on gut hormone access. Lastly, there was limited research found on BPD-DS. BPD-DS is a much more drastic surgery than the other surgeries mentioned, leading to lifelong detrimental effects.

The information from the articles gathered for this paper is displayed in Table 1.

**Table 1:** Summarization of publications used in the study per PRISMA [32] T2DM: Type 2 diabetes mellitus

	Author	Country	Design & Study Population	Findings	Conclusion
1	Benedix et al., 2011 [58]	Germany	Cohort (n = 65)	Fasting serum insulin decreased significantly for sleeve gastrectomy and Roux-en-Y gastric bypass. Sleeve gastrectomy patients had an increase in salivary ghrelin, higher than baseline, whereas Roux-en-Y gastric bypass had a further decrease. The adjustable gastric band had no changes to ghrelin levels after 12 months. An increase in sleeve gastrectomy and Roux-en-Y gastric bypass of high molecular weight adiponectin was seen at 12 months 104% and 105%, respectively.	Weight loss is superior in sleeve gastrectomy compared to adjustable gastric band but comparable to Roux-en-Y gastric bypass 12 months after surgery. A decline in fasting insulin was only seen in sleeve gastrectomy and Roux-en-Y gastric bypass patients, while ghrelin varied between the three procedures. Also, high molecular weight adiponectin was significantly increased in sleeve gastrectomy and Roux-en-Y gastric bypass compared to adjustable gastric band.
2	Bunt et al., 2017 [63]	USA	Prospective study (n = 18)	Insulin low-carbohydrate mixed meal and C-peptide were lower in adjustable gastric band patients with no change in estimated hepatic insulin extraction, whereas in Roux-en-Y gastric bypass fasting insulin and glucose were decreased Still, no C-peptide and hepatic insulin extraction increased during Insulin low-carbohydrate mixed meal and at fasting. In Roux-en-Y gastric bypass, early prandial insulin and glucose responses significantly increased.	Different changes in plasma insulin, gut hormone responses to Insulin low-carbohydrate mixed meal, and insulin were strongly apparent between Roux-en-Y gastric bypass and adjustable gastric band. The changes, such as increased insulin response and altered trajectory of a glucagon-like peptide 1, show an earlier improvement of glycemia observed with Roux-en-Y gastric bypass surgery versus adjustable gastric band surgery.
3	Johansson et al., 2010 [62]	Sweden	Experimental study (n = 10)	Despite higher BMI, fasting insulin and glucose decreased in the biliopancreatic diversion with duodenal switch postprandial proinsulin, glucose, and insulin did not differ between biliopancreatic diversion with duodenal switch and control. Insulin and proinsulin concentrations were lower in the later phase of biliopancreatic diversion with duodenal switch.	Biliopancreatic diversion with duodenal switch provokes a significant weight loss and almost normalizes postprandial responses of proinsulin, insulin, and glucose with a significant decrease in triglycerides.
4	Christou et al., 2015 [52]	Canada	Prospective cohort (n = 36)	Plasma ghrelin concentrations were significantly lower in patients following Roux-en-Y gastric bypass than lean controls. Ghrelin levels pre or postprandially were not different between patients who had a successful weight loss or those who achieved a less-than-ideal weight loss. There was a strong inverse correlation between preprandial ghrelin concentrations and the current or pre-operative BMI.	Weight loss failure after Roux-en-Y gastric bypass does not correlate with ghrelin concentrations pre- or post-prandially. Ghrelin concentrations were inversely proportional to BMI and were not associated with satiety.
5	Xu et al., 2019 [53]	China	Meta-analysis (n = 325)	Ghrelin concentrations had an increasing trend. Ghrelin concentrations decreased short-term (≤ 3 months) and increased long-term (> 3 months) after Roux-en-Y gastric bypass. Meta-regression showed that gastric pouch volume, biliopancreatic limb length, and alimentary limb length were not associated with changes in ghrelin concentrations.	Ghrelin concentrations decreased short-term (≤ 3 months) and increased long-term (> 3 months) after Roux-en-Y gastric bypass.
6	Dadan et al., 2009 [39]	Poland	Cohort study (n = 18)	On the first day after surgery, adjustable gastric band and Roux-en-Y gastric bypass ghrelin levels decrease. Seven days after surgery, ghrelin levels increased for an adjustable gastric band and Roux-en-Y gastric bypass. At the one-month mark, adjustable gastric band ghrelin levels were 79.55 pg/ml, and Roux-en-Y gastric bypass was 92.8 pg/ml. However, at the three-month mark, adjustable gastric band levels were similar to controls, while significantly lower than control and adjustable gastric band.	Overall, Roux-en-Y gastric bypass patients had lower gastrin levels post-surgery than controls, while adjustable gastric band patients had increased ghrelin levels post-surgery. However, one month after surgery, ghrelin levels increased in Roux-en-Y gastric bypass gastric bypass, and then three months after surgery, ghrelin decreased below the control group significantly.
7	Dixon et al., 2005 [57]	Australia	Experimental study (n = 17)	There was no difference in insulin, glucose, leptin, or ghrelin, which is optimal in reduced restriction after an adjustable gastric band. ABG subjects had higher ghrelin levels and lower glucose, insulin, and leptin levels than the controls.	Plasma leptin, insulin, and ghrelin were unrelated to the effect of satiety and showed orexigenic compensatory changes. The mechanism of satiety is not fully understood and needs to be explored as it may lead to the understanding of energy homeostasis.
8	Edwards et al., 2010 [70]	USA	Cohort study (n = 16)	Patients who lost 10-20% of excess body weight had a 1.9-fold decrease in resistin level, while those who lost less than 10% had a 2.4-fold increase. Leptin levels in patients increased in patients who lost less than 10%. Overall, there was a decrease in resistin patients who underwent Roux-en-Y gastric bypass. Mean leptin expression after adjustable gastric band increased 1.6-fold while decreasing 2.7-fold in Roux-en-Y gastric bypass patients.	After Roux-en-Y gastric bypass, there is a downregulation of leptin and resistin gene expression, allowing for the normalization of insulin resistance in obesity. Roux-en-Y gastric bypass specifically, a significant downregulation of leptin and resistin gene proportional to greater excess body weight loss while there was no change in the adjustable gastric band.
9	Fruhbeck et a., 2011 [68]	Spain	Cohort study (n = 13)	There was a significant decrease in pre- and post-surgery glucose and insulin levels in adjustable gastric band patients and Nissen fundoplication. There was also an increase in leptin levels following adjustable gastric band surgery and a slight decrease after Nissen fundoplication surgery.	There were significant alterations in glucose, insulin, and leptin levels following adjustable gastric band surgery. The increase in leptin levels may be the reason why patients have earlier satiety signaling due to the trigger of increased leptin levels.
10	Zarshenas et al., 2021 [67]	Australia	Retrospective cohort study (n = 73)	Significantly higher weight loss was observed in the adjustable gastric band group compared to the Roux-en-Y gastric bypass group only at the one-year follow-up. Both groups showed significantly improved insulin and fasting blood glucose at two years.	Weight loss was similar after the adjustable gastric band and Roux-en-Y gastric bypass, but the adjustable gastric band was seen to have a higher rate of gastrointestinal adverse effects.
11	Gelisgen et al., 2011 [36]	Turkey	Cohort study (n = 21)	Ghrelin concentrations (plasma and fundic acylated) were significantly lower in severely obese patients than in non-obese controls. Ghrelin was increased dramatically in severely obese patients six months following an adjustable gastric band.	Significant weight was lost and continued after the adjustable gastric band despite a significant increase in fundic acylated and plasma ghrelin. The relationship between weight loss through surgery and ghrelin needs to be explored.
12	Elias et al., 2022 [33]	Sweden	Prospective study (n = 28)	Following biliopancreatic diversion with duodenal switch, patients lost an average of 66.1% of their excess BMI. Leptin concentrations were halved. Insulin and glucose were also reduced. While ghrelin and motilin were reduced, they were accompanied by significant peaks 30 minutes before a meal. Glucose-dependent insulinotropic polypeptide concentrations were reduced, while glucagon-like peptide 1 and Peptide YY responses were significantly increased.	Biliopancreatic diversion with duodenal switch reduces leptin and ghrelin levels while increasing glucagon-like peptide 1 and Peptide YY.
13	Kashyap et al., 2009 [77]	USA	Cohort study (n = 16)	One week following surgery, both adjustable gastric band, and Roux-en-Y gastric bypass groups showed a similar reduction in fasting glucose and similar weight loss. Insulin sensitivity improved with Roux-en-Y gastric bypass. Following Roux-en-Y gastric bypass, insulin low-carbohydrate mixed meal significantly increased glucagon-like peptide 1 level, insulin secretion, and β-cell sensitivity to glucose.	Roux-en-Y gastric bypass, compared to an adjustable gastric band, elicits a rapid improvement in glucose regulation, which is partnered with enhanced β-cell sensitivity to glucose and insulin sensitivity due to the incretin effect.
14	Korner et al., 2009 [45]	USA	Longitudinal study (n = 43)	One year following the Roux-en-Y gastric bypass, there was more significant weight loss than an adjustable gastric band, but the final BMI was similar. Peptide YY concentrations 52 weeks after the Roux-en-Y gastric bypass were greater than the adjustable gastric band. Postprandial glucagon-like peptide 1 concentrations were threefold greater following Roux-en-Y gastric bypass than adjustable gastric band. Ghrelin concentrations increased following the adjustable gastric band but had a decreasing trend after the Roux-en-Y gastric bypass.	Varying concentrations of gut hormones may promote more significant weight loss after Roux-en-Y gastric bypass compared to adjustable gastric band and insulin sensitivity. Still, decreases in T3 and leptin may limit weight loss.
15	Kotidis et al., 2006 [34]	Greece	Experimental study (n = 13)	After biliopancreatic diversion with duodenal switch, BMI significantly decreased, from 59.15 kg/m^2^ to 32.91 kg/m^2^. Serum fasting ghrelin concentrations decreased, serum leptin concentrations decreased, and adiponectin concentration increased.	Biliopancreatic diversion with duodenal switch is correlated with a significant decrease in serum ghrelin levels. Leptin decreased insignificantly, and adiponectin increased insignificantly.
16	Auguet et al., 2022 [80]	Switzerland	Prospective cohort study (n = 65)	At 12 months postoperatively, weight, BMI, fasting glucose, insulin, HbA1c concentrations, HOMA2-IR, LDL-C, C-peptide, triglyceride concentrations, AST, ALT, GGT, ALP, leptin, PAI-1, resistin, lipocalin, TNF-a, and IL-1B were decreased, and HDL-C concentrations, adiponectin, IL-10, and IL-8 were increased.	This study concludes that baseline adipocytokines may help predict metabolic changes after bariatric surgery.
17	Perakakis et al., 2019 [59]	USA	Longitudinal cohort study (n = 42)	Study 1 found that glucagon-like peptide 1 and glucagon-like peptide 2 levels were reduced following Roux-en-Y gastric bypass compared to adjustable gastric band. 6 months following the adjustable gastric band, subjects had minor changes in circulating levels of glucagon-like peptide 1 and Peptide YY. However, RYBG patients had significantly increased peptide YY and glucagon-like peptide 1. In Study 2, fasting and postprandial glucose and insulin were significantly reduced following sleeve gastrectomy and Roux-en-Y gastric bypass. Peptide YY preprandial concentrations were higher for twelve months. In the sleeve gastrectomy group, ghrelin concentrations were significantly lower.	Their study concluded that postprandial oxyntomodulin and glicentin changes were strong predictors of weight loss following bariatric surgery, possibly due to the regulation of satiety.
18	Shak et al., 2007 [60]	USA	Prospective cohort study (n = 24)	Leptin concentration decreased from baseline 12 months following adjustable gastric band surgery. Plasma concentrations of total and acylated ghrelin, insulin, glucagon-like peptide 1, gastrin, glucose-dependent insulinotropic polypeptide, and pepsinogen I were unchanged from surgery.	Adjustable gastric band results in drastic % excess weight loss with a proportional decrease in leptin concentrations.
19	Tsouristakis et al., 2019 [41]	USA	Prospective cohort study (n = 56)	While postprandial glucose stayed significantly lower than baseline following Roux-en-Y gastric bypass, the adjustable gastric band had an initial significant decrease in postprandial glucose. Both adjustable gastric band and Roux-en-Y gastric bypass had significant increases in ghrelin at Year 2, but the rise in ghrelin correlated to the percent weight loss. Fasting and postprandial peptide YY increased following the Roux-en-Y gastric bypass but not after the adjustable gastric band. Postprandial concentrations of glucagon-like peptide 1 were considerably high only in the Roux-en-. Leptin levels decreased following both surgeries but were negatively associated with percent weight loss.	Over time, significant changes in the gut hormones such as Peptide YY, glucagon-like peptide 1, insulin, ghrelin, and leptin may reflect the gut enteroplasticity following Roux-en-Y gastric bypass.
20	Dirksen et al., 2013 [51]	Denmark	Prospective cohort study (n = 33)	Hunger was more suppressed in good weight loss than in poor weight loss response to the multiple-meal test. Good responders had a more significant release of glucagon-like peptide 1 and suppressing ghrelin, whereas postprandial CCK secretion was more significant in poor responders. Peptide YY, PP, neurotensin, and total bile acid did not differ between groups.	The alteration between gut hormones between good responders and poor responders of Roux-en-Y gastric bypass indicates that hormones may play a part in why weight may be difficult to lose or keep off for some patients.
21	Michaud et al., 2017 [35]	Canada	Experimental study (n = 27)	There was no normalization of fasting and postprandial glucose concentrations in those with T2DM compared to control. Postprandial plasma pancreatic peptides were significantly increased in T2DM patients compared to controls. Fasting and postprandial glucagon-like peptide 1 concentrations were increased significantly after sleeve gastrectomy. Fasting and postprandial ghrelin concentrations were reduced significantly for both groups after surgeries. Postprandial glucose concentrations were significantly decreased in biliopancreatic diversion with duodenal switch patients compared to Sleeve gastrectomy. Total ghrelin concentrations were significantly increased in biliopancreatic diversion with duodenal switch patients.	Hepatic insulin sensitivity and β-cell function significantly improved Three days after Sleeve gastrectomy and biliopancreatic diversion with duodenal switch. This suggests the role of food restriction and its effect as an early anti-diabetic effect following bariatric surgery. Moreover, excluding the duodenum and proximal jejunum can aid caloric restriction early after biliopancreatic diversion with duodenal switch.
22	Svane et al., 2016 [74]	Denmark	Prospective cohort study (n = 9); Cross-over study (n = 12)	Study 1: There was a 35% decrease in food intake following Roux-en-Y gastric bypass. Before surgery, blockage of the glucagon-like peptide 1 receptor increased food intake, but this was not seen postoperatively, while secretion of Peptide YY was significantly increased. Study 2: Blockage of the glucagon-like peptide 1 receptor and DPP-4 inhibitor lowered Peptide YY (3-36) and increased food intake by 20% following Roux-en-Y gastric bypass, but no effect was seen alone.	Blockage of both glucagon-like peptide 1 and Peptide YY increased food intake following bariatric surgery, which supports hormones playing a role in the decreased food intake following surgery.
23	Arakawa et al., 2020 [56]	USA	Prospective cohort study (n = 59)	The Roux-en-Y gastric bypass group had more significant weight loss than the Sleeve gastrectomy group at 52 weeks. Significant postprandial Peptide YY increase compared to baseline after Roux-en-Y gastric bypass. Peptide YY increased after sleeve gastrectomy at 26 weeks, but significance was not maintained at 52 weeks. Glucagon-like peptide 1 after sleeve gastrectomy was higher than Roux-en-Y gastric bypass at 26 weeks but not maintained at 52 weeks. Roux-en-Y gastric bypass glucagon-like peptide 1 persisted at 52 weeks. Only after sleeve gastrectomy did ghrelin levels decrease compared to baseline.	Characteristics of hormone changes following sleeve gastrectomy and Roux-en-Y gastric bypass may be a possible mechanism for greater efficacy in Roux-en-Y gastric bypass than sleeve gastrectomy.
24	Shimizu et al., 2017 [72]	Japan	Prospective cohort study (n = 10)	Sleeve gastrectomy patients showed a sharp decrease in leptin levels with a significant increase in FGF-19 1 month after surgery. At one month following surgery, adiponectin concentrations show an increasing trend, significantly increasing at six months.	Sleeve gastrectomy surgery increased FGF-19 right after surgery, slowly corrected obesity-related inflammation, and improved adipocytokine secretion.
25	Gomez-Martin et al., 2017 [64]	Spain	Prospective cohort study (n = 60).	Adiponectin increased after surgery, markedly after Roux-en-Y gastric bypass than sleeve gastrectomy. Adiponectin did not correlate with carotid intima-media thickness.	Roux-en-Y gastric bypass leads to a higher increase in adiponectin, which correlates with increased sex hormone binding globulin and reduced fasting insulin and insulin resistance. There was no association with lipid profiles, carotid intimal medial, or blood pressure.
26	Kalinowski et al., 2016 [43]	Poland	Prospective cohort study (n = 72)	The percentage of excess weight loss after Roux-en-Y gastric bypass at 12 months was 67.6% after sleeve gastrectomy and 64.2%. Fasting ghrelin concentrations decreased one-month following sleeve gastrectomy and were maintained until 12 months. Fasting ghrelin increased 12 months after the Roux-en-Y gastric bypass.	Roux-en-Y gastric bypass and sleeve gastrectomy have comparable results for weight loss and glucose metabolism improvement. Ghrelin levels decrease after sleeve gastrectomy but increase after Roux-en-Y gastric bypass. Weight loss in both groups seems to be influenced by multiple factors.
27	Lampropoulos et al., 2022 [55]	Greece	Cross-sectional study (n = 32).	Standard liquid mix meal ingestion did not significantly change fast gastrointestinal hormone concentrations following Sleeve gastrectomy or BDP/Roux-en-Y gastric bypass long limb. In both surgeries, weight maintenance and weight regain groups did not have different fasting ghrelin concentrations. Standard liquid mix meal ingestion significantly suppresses ghrelin levels in weight maintenance Sleeve gastrectomy patients. Lower postprandial levels were seen in weight maintenance patients in both surgeries, whereas an increase in fasting and postprandial levels was positively correlated with BMI in weight regain groups.	Long-term weight regain after bariatric surgery is not associated with unfavorable GI hormone secretion pattern.
28	Lee et al., 2011 [46]	Taiwan	Prospective cohort study (n = 32)	T2DM remission was attained in 81% Roux-en-Y gastric bypass and 19% sleeve gastrectomy patients two years after surgery. Patients with Roux-en-Y gastric bypass lost more weight and had lower levels of HbA1c, glucose, insulin resistance, and smaller waist circumference than sleeve gastrectomy patients.	Roux-en-Y gastric bypass and sleeve gastrectomy strongly affect the hindgut, but due to the duodenal exclusion effect, Roux-en-Y gastric bypass significantly impacts CCK levels. Sleeve gastrectomy patients have lower acyl and des-acyl ghrelin concentrations but more significant resistin levels than Roux-en-Y gastric bypass patients.
29	Rigamonti et al., 2016 [75]	Italy	Prospective study (n = 10)	There was no difference in glucagon-like peptide-2 release following fast or slow feeding pre- and post-sleeve gastrectomy. Peptide YY concentrations increased after both fast and slow feeding post-sleeve gastrectomy. Glucagon-like peptide 1 postprandial levels did not change before or after surgery. Plasma levels of Peptide YY postprandially were higher post-sleeve gastrectomy. Fast feeding leads to a higher satiety level than slow feeding before and after surgery.	This study concluded that there are non-significant contributions of anorexigenic gut peptides in response to Sleeve gastrectomy surgery.
30	Yousseif et al., 2013 [48]	United Kingdom	Prospective study (n = 18)	Compared to Roux-en-Y gastric bypass, sleeve gastrectomy decreased acyl ghrelin concentration fasting and postprandially. In both groups, active glucagon-like peptide 1 and Peptide YY (3-36) increased postoperatively, with Roux-en-Y gastric bypass having greater concentrations. Roux-en-Y gastric bypass was more likely to suppress hunger compared to sleeve gastrectomy.	While sleeve gastrectomy and Roux-en-Y gastric bypass had different gut and hormone profiles alterations, both produced comparable satiety and weight loss.
31	Gu et al., 2020 [49]	China	Meta-analysis (n = 1843)	Fasting acyl-ghrelin fasting Peptide YY was significantly lower in the sleeve gastrectomy group compared to Roux-en-Y gastric bypass. After Roux-en-Y gastric bypass, fasting Peptide YY was significantly higher than baseline, with a significant increase in fasting ghrelin concentrations	Ghrelin decreases significantly following sleeve gastrectomy, and Peptide YY increases significantly following Roux-en-Y gastric bypass. The interplay between these hormones and their respective surgery is complicated, and further experiments are needed.
32	Hedberg al., 2010 [73]	Sweden	Experimental study (n = 20)	Fasting Peptide YY concentrations were significantly higher in biliopancreatic diversion with duodenal switch patients than in controls and after the postprandial meal.	Despite preserving the pylorus in biliopancreatic diversion with duodenal switch, stomach emptying is quicker than controls. Fasting Peptide YY concentrations were elevated compared to controls. Postprandial Peptide YY was also significantly increased.
33	McCarty et al., 2019 [40]	USA	Meta-analysis (n = 653)	Before sleeve gastrectomy, the average BMI decreased. Ghrelin concentrations decreased while glucagon-like peptide 1 and YY increased following sleeve gastrectomy. Glucose-dependent insulinotropic polypeptide concentrations did not significantly change following sleeve gastrectomy.	Fasting ghrelin concentrations decreased while glucagon-like peptide 1 and peptide YY increased following sleeve gastrectomy; fasting glucose-dependent insulinotropic polypeptide concentrations remained unchanged.
34	Min et al., 2019 [79]	UK	Prospective cohort study (n = 16)	At 1 and 6 months following sleeve gastrectomy, there was a significant increase in glucagon-like peptide 1 secretion, but this was not maintained at four years. In biliopancreatic diversity, there was a reduction in HbA1c and an increase in glucagon-like peptide 1 response seen at seven years post surgery.	Following sleeve gastrectomy, there was an increase in glucagon-like peptide 1 concentration, but it was not maintained at four years. There was also an increase in glucose-dependent insulinotropic polypeptide response in parallel with improved glycemic control.
35	Navarro-Garcia et al., 2020 [50]	Spain	Prospective cohort study (n = 54)	There were no significant differences in acylated ghrelin levels between sleeve gastrectomy and Roux-en-Y gastric bypass. Both procedures saw an increase on day 5 followed by a decrease, and then the levels gradually increased at 12 months to levels higher than baseline. Despite increased ghrelin levels, there was no effect on weight loss, achieving 30% weight loss.	Compared to baseline, there was an increase in fast acylated ghrelin concentrations after one year following Sleeve gastrectomy and Roux-en-Y gastric bypass.
36	Ramon et al., 2012 [54]	Spain	Prospective cohort study (n = 15)	There was a significant decrease in insulin and plasma glucose levels following Roux-en-Y gastric bypass and sleeve gastrectomy. After the Roux-en-Y gastric bypass, there was a significant decrease in fasting and postprandial leptin concentrations and a significant increase in glucagon-like peptide 1 following a meal test. There was a significant decrease in fasting ghrelin concentrations after sleeve gastrectomy.	Overall, both sleeve gastrectomy and Roux-en-Y gastric bypass significantly improve glucose homeostasis. While glucagon-like peptide 1 and Peptide YY levels were increased similarly in sleeve gastrectomy and Roux-en-Y gastric bypass, only sleeve gastrectomy patients had decreased fasting and postprandial ghrelin levels.
37	Yang et al., 2018 [47]	China	Prospective cohort study (n = 20)	One month after surgery, fasting plasma glucose decreased significantly and remained stable in sleeve gastrectomy and Roux-en-Y gastric bypass. Peptide YY and fasting glucagon-like peptide 1 concentrations increased significantly in both groups but more after Roux-en-Y gastric bypass. Ghrelin concentrations were not significantly different following Roux-en-Y gastric bypass but were significantly decreased after sleeve gastrectomy.	Roux-en-Y gastric bypass and sleeve gastrectomy significantly change gastrointestinal hormones, decreasing plasma glucose and glucagon and increasing peptide YY and glucagon-like peptide 1. Sleeve gastrectomy patients had decreased ghrelin following surgery. Only Roux-en-Y gastric bypass had reduced glucose-dependent insulinotropic polypeptide concentrations.
38	Svane et al., 2019 [38]	Denmark	Cross-sectional study (n = 36)	Ingested glucose dissemination occurred faster after sleeve gastrectomy and Roux-en-Y gastric bypass than the controls, and the peak appearance rate was 23% higher after sleeve gastrectomy and 64% higher after Roux-en-Y gastric bypass. There were larger increases in glucagon-like peptide 1, insulin, CCK, and Peptide YY secretions after Roux-en-Y gastric bypass, whereas ghrelin concentrations were lower after sleeve gastrectomy than Roux-en-Y gastric bypass and controls.	Postprandial glucose and gastroenteric pancreatic hormone secretions differ between sleeve gastrectomy and Roux-en-Y gastric bypass.
39	Peterli et al., 2012 [37]	Switzerland	Prospective cohort study (n = 23)	Following sleeve gastrectomy and Roux-en-Y gastric bypass, patients had an increase in postprandial plasma Peptide YY and glucagon-like peptide 1 concentration. Ghrelin concentrations approached pre-operative levels 12 months after Roux-en-Y gastric bypass, whereas ghrelin levels were steadily attenuated following sleeve gastrectomy.	Bariatric surgery alters the balance between the foregut and the hindgut. Understanding this interplay may be essential to understanding the underlying mechanism of bariatric surgery.
40	Dardzińska et al 2018 [42]	Poland	Prospective cohort study (n = 23)	Acyl and desacyl ghrelin concentrations and acyl/desacyl ratio did not change after six and 12 months in both adjustable gastric band and Roux-en-Y gastric bypass groups. In sleeve gastrectomy, there was a significant decrease in pre- and postprandial desacyl levels and an increase in fasting acyl/desacyl ratio after six and 12 months.	There is a reduction in body weight and BMI after bariatric surgery. Additionally, desacyl ghrelin and acyl ghrelin levels are unaffected one year after adjustable gastric band and Roux-en-Y gastric bypass surgery but decrease one year after Sleeve gastrectomy surgery.
41	Seridi et al., 2018 [71]	China	Experimental study (n=27)	Leptin significantly decreased one week following Roux-en-Y gastric bypass surgery and fell below the level in control subjects after three months.	Leptin levels decrease significantly following Roux-en-Y gastric bypass, and this may be linked to the normalization of microbial-derived metabolites.
42	Adami et al., 2016 [69]	Italy	Prospective cohort study (n = 30)	Serum leptin concentrations decreased after the first year and remained unchanged long-term in both patients. A significant increase in serum adiponectin concentration was observed only in remitter patients. Serum leptin concentration mean values were positively correlated with BMI before and following biliopancreatic diversion with duodenal switch, and adiponectin levels were positively associated with the postoperative acute insulin response data.	Improvement of cytokine production, seen via increased adiponectin concentrations, may play a role in T2DM remission following bariatric surgery.
43	Woelnerhanssen et al., 2011 [66]	Switzerland	Prospective cohort study (n = 23)	There was a significant decrease in body weight in both Roux-en-Y gastric bypass and sleeve gastrectomy. Leptin concentrations decreased by almost 50% in 1 week following both surgeries and continued to decline until 12 months. Adiponectin also progressively increased following both surgeries.	Roux-en-Y gastric bypass and sleeve gastrectomy leads to significant weight loss paired with metabolic syndrome resolution. Leptin concentrations decreased while adiponectin increased, paralleled by improved insulin sensitivity.
44	Cazzo et al., 2018 [76]	Brazil	Prospective cohort study (n = 12)	There were significant changes in BMI, HbA1c, total cholesterol, LDL-C, and postprandial glucagon-like peptide-2.	Different satiety regulation components correlate with the postprandial concentrations of glucagon-like peptide 1 (hunger) and glucagon-like peptide-2 (satiation) a year after modified biliopancreatic diversion with duodenal switch.
45	Karamanakos et al., 2008 [44]	Greece	Prospective cohort study (n = 16)	BMI and body weight decreased significantly after sleeve gastrectomy and Roux-en-Y gastric bypass. Fasting ghrelin levels non-significantly changed after Roux-en-Y gastric bypass nor postprandially. Fasting ghrelin levels significantly decreased after sleeve gastrectomy. Fasting Peptide YY levels increased significantly after Roux-en-Y gastric bypass and sleeve gastrectomy.	While Peptide YY levels increased significantly in Roux-en-Y gastric bypass and sleeve gastrectomy patients, sleeve gastrectomy had significantly reduced ghrelin levels associated with higher appetite suppression, leading to more excess weight loss.
46	Cazzo et al., 2017 [61]	Brazil	Prospective cohort study (n = 6)	Standard insulin low-carbohydrate mixed meal elicited a significant increase in glucagon-like peptide-2 levels following biliopancreatic diversion with duodenal switch at 15 to 60 minutes. Following this trend, glucagon-like peptide-2 gradually decreased close to the baseline.	There is a significant increase in glucagon-like peptide-2 levels following biliopancreatic diversion with duodenal switch. The clinical implications of this finding are not fully understood.
47	Hollanda et al., 2014 [78]	Spain	Cross-sectional study (n = 30)	Failed weight loss patients had less suppression of ghrelin and a lesser increase in glucagon-like peptide 1 but not glucagon-like peptide-2 and Peptide YY in response to meal intake.	Some gastrointestinal hormones may have a role in mediating successful weight loss.
48	Swarbrick et al., 2008 [65]	USA	Experimental study (n=19)	Roux-en-Y gastric bypass surgery reduced fasting insulin, fat mass, and glucose concentration. At one and three months following surgery, there was a reduction in fasting pancreatic polypeptide and glucagon levels, respectively.	Adipocytes and pancreatic hormones may impact the long-term resolution of insulin resistance following Roux-en-Y gastric bypass.

## Conclusions

Plasma ghrelin concentrations are mainly regulated by food intake. It is thought that ghrelin may be decreased by the hyperinsulinemia brought on by bariatric surgery. The type of surgery strongly influences fasting ghrelin concentrations, but studies still show some conflicts as fasting ghrelin concentrations increase with weight loss. These concentrations are different even within surgeries but are not the only factor contributing to surgery's success. The decreased insulin observed following the surgeries, while indicating improved insulin sensitivity in T2DM, may assist in the continued weight loss following surgery. RYGB overall showed a more significant insulin reduction than the other surgeries. This correlates with RYGB being the second most successful surgery, but there is no comparison to BPD-DS, which hinders the interpretation of these results. Leptin concentrations decreased following most surgeries, possibly because of the correction to leptin resistance that is increased in obesity. RYGB also produced the lowest concentration of leptin, excluding comparison to BPD-DS. One study has results that suggest leptin concentrations correlated with weight loss. PYY functions in gastric emptying and is generally increased following bariatric surgery. This was consistent in BPD-DS, but only postprandial PYY showed a significant increase in RYGB, SG, and AGB. GLP-1 influences insulin release, which means it can also influence weight loss. Overall, RYGB patients indicated a more significant increase in GLP-1 concentrations. Adiponectin has been shown to prevent obesity and increase during weight loss, and this was observed in most surgeries. Like with most hormones, there was a more significant increase in adiponectin following RYGB, but no difference was seen between SG and BPD-DS.

More research needs to be done to compare BPD-DS and RYGB. In our search, RYGB has earned its spot as a highly successful surgery as associated hormone concentrations showed to be significantly influenced, correlating with significant weight loss. There are many factors to consider, but hormones affect our eating behaviors and metabolism. BPD-DS theoretically should alter hormones to a greater degree due to the extent of surgery, but despite having the lowest failure rate, it has the highest complication rate. According to hormone profiles, RYGB does not have the same complication rates and may be the best surgery for weight loss.
